# Non-invasive measurements of pulse pressure variation and stroke volume variation in anesthetized patients using the Nexfin blood pressure monitor

**DOI:** 10.1007/s10877-015-9759-7

**Published:** 2015-08-29

**Authors:** Jurre Stens, Jeroen Oeben, Ab A. Van Dusseldorp, Christa Boer

**Affiliations:** Department of Anesthesiology, Institute for Cardiovascular Research, VU University Medical Center, De Boelelaan 1117, 1081 HV Amsterdam, The Netherlands

**Keywords:** Hemodynamic, Non-invasive monitoring, Blood pressure, Cardiac output, Anesthesia, Fluid challenge

## Abstract

Nexfin beat-to-beat arterial blood pressure monitoring enables continuous assessment of hemodynamic indices like cardiac index (CI), pulse pressure variation (PPV) and stroke volume variation (SVV) in the perioperative setting. In this study we investigated whether Nexfin adequately reflects alterations in these hemodynamic parameters during a provoked fluid shift in anesthetized and mechanically ventilated patients. The study included 54 patients undergoing non-thoracic surgery with positive pressure mechanical ventilation. The provoked fluid shift comprised 15° Trendelenburg positioning, and fluid responsiveness was defined as a concomitant increase in stroke volume (SV) >10 %. Nexfin blood pressure measurements were performed during supine steady state, Trendelenburg and supine repositioning. Hemodynamic parameters included arterial blood pressure (MAP), CI, PPV and SVV. Trendelenburg positioning did not affect MAP or CI, but induced a decrease in PPV and SVV by 3.3 ± 2.8 and 3.4 ± 2.7 %, respectively. PPV and SVV returned back to baseline values after repositioning of the patient to baseline. Bland–Altman analysis of SVV and PPV showed a bias of −0.3 ± 3.0 % with limits of agreement ranging from −5.6 to 6.2 %. The SVV was more superior in predicting fluid responsiveness (AUC 0.728) than the PVV (AUC 0.636), respectively. The median bias between PPV and SVV was different for patients younger [−1.5 % (−3 to 0)] or older [+2 % (0–4.75)] than 55 years (*P* < 0.001), while there were no gender differences in the bias between PPV and SVV. The Nexfin monitor adequately reflects alterations in PPV and SVV during a provoked fluid shift, but the level of agreement between PPV and SVV was low. The SVV tended to be superior over PPV or Ea_dyn_ in predicting fluid responsiveness in our population.

## Introduction

Pulse pressure variation (PPV) and stroke volume variation (SVV) are increasingly used to monitor hemodynamic changes and guide fluid management in non-cardiac surgery or during intensive care unit admission in mechanically ventilated patients [1, 2]. In particular, both dynamic indices have a higher sensitivity and specificity for the prediction of fluid responsiveness in critically ill patients than stroke volume or cardiac index [3].

Monitoring of hemodynamic indices in the perioperative setting, like the PPV or cardiac index, requires devices that allow beat-to-beat registration of hemodynamic parameters. The introduction of non-invasive continuous blood pressure measurement devices in the perioperative setting, like the Nexfin^®^ or CNAP-500^®^, facilitates monitoring of these indices in patients without an indication for an intra-arterial line [4, 5]. In particular, the Nexfin hemodynamic monitor is increasingly used in the perioperative setting and validated for non-invasive measurements of arterial blood pressure [6–9], cardiac index (CI) [10–12] and systolic pressure variation [13, 14]. Using a finger cuff, Nexfin arterial blood pressure measurements are based on the volume-clamp method with an internal physiologic calibration procedure [6–8]. The Nexfin device assesses the PPV based on its continuous arterial blood pressure signal, and uses an automated application of arterial pulse wave algorithm to calculate the SVV [2–4]. This algorithm is based on the input of age, gender and body surface area to estimate aorta compliance [11]. Specific pulse contour analysis for input in the algorithm will subsequently guide the determination of stroke volume (SV) a cardiac index [11].

The clinical applicability of Nexfin PPV and SVV for monitoring of intraoperative fluid shifts has only scarcely been investigated and sometimes showed difficulties in rapidly reflecting fluid challenge induced changes in cardiac index [14, 15]. In the present study we therefore investigated whether Nexfin monitoring can be used to determine the effect of a mild provoked fluid shift by Trendelenburg positioning in mechanically ventilated patients on PPV and SVV, and the level of agreement between both indices.

## Materials and methods

### Patient population

This observational clinical study was performed in the VU University Medical Centre. The Human Subjects Committee of VU University Medical Centre approved this study (METc VUmc 2013/087) and patients provided informed consent. Included patients underwent elective surgery under general anesthesia with positive pressure mechanical ventilation. All measurements took place between endotracheal intubation and the first surgical incision during a steady state period following anesthesia induction. Patients aging 20–54 and 55–75 years were included based on an a priori determined cut-off value for age. Exclusion criteria were pregnancy, diabetes mellitus, hypertension, aortic stenosis, chronic heart failure, cardiac arrhythmias, peripheral vascular disease or a body mass index (BMI) exceeding 35 kg/m^2^. The study ended after repositioning to supine steady state following Trendelenburg positioning.

### Anesthesia and mechanical ventilation

The anesthetic procedure was not standardized to adhere to routine anesthesia practice, and included general anesthesia using propofol and/or sevoflurane (AbbVie BV, Hoofddorp, The Netherlands). Vasopressors during anesthesia induction were used upon discretion of the anesthesiologist. After induction of anesthesia, patients were endotracheally intubated and mechanically ventilated with a positive end-expiratory pressure of 5 mmHg, a tidal volume of 8 ml/kg and a variable, individual ventilation frequency among patients.

### Non-invasive arterial blood pressure measurements

Beat-to-beat non-invasive arterial blood pressure measurements were performed using the Nexfin^CC^-monitor (Edwards Lifesciences, Amsterdam, The Netherlands). Since multiple studies already demonstrated acceptable accuracies of Nexfin cardiac index measurements compared to gold standard methods like thermodilution or Doppler echocardiography, we used the Nexfin without comparing it to a gold standard [6–8, 10–17].

Briefly, arterial blood pressure waveforms are derived by optical plethysmography using a finger cuff. The finger cuff size was chosen based on the measured circumference of the middle phalanx of the third digital. A feedback system controls the pressure in the finger cuff, such that finger artery diameter is kept at a constant volume according to the volume-clamp method. A transfer function model is applied to reconstruct the brachial arterial blood pressure waveform from the finger arterial pressure and to correct for pressure differences due to a resistance of flow [11]. The primary measurement objective of the Nexfin^CC^-monitor is arterial blood pressure, enabling to subsequent determination of stroke volume, cardiac index, and stroke volume variation using the Nexfin CO-trek-algorithm and blood pressure based calculation of pulse pressure variation [11]. Stroke volume and cardiac index calculation require the entry of patient demographics, including patient age, gender and body surface area as described above. A built-in expert system for calibration (Physiocal, BMEYE BV, Amsterdam, The Netherlands) adjusts the cuff to determine a proper volume clamp set point. A heart reference system (HRS) was positioned on the axillary line of the thorax, at the level of the 4th intercostal space and is used to compensate for hydrostatic differences between heart and finger cuff level.

### Study procedures

After endotracheal intubation, patients were positioned in supine position and the heart reference system was positioned on the axillary line of the thorax, at the level of the 4th intercostal space. Demographic variables included body weight, length, age and gender, which were entered into the Nexfin^CC^-monitor for calculation of stroke volume and cardiac index. A mark was set on the monitor, and hemodynamic steady state measurements were initiated and continued for 4 min. Subsequently, the table was adjusted to Trendelenburg position (15°) and another mark was set at the monitor. Trendelenburg positioning was initiated during a steady state period following anesthesia induction and no vasopressors were administered during the study period. The measurement in Trendelenburg position continued for 2 min, and the table was subsequently returned to a neutral position. A new mark was set and the measurement continued for another minute in neutral position, which marked the end of the measurement procedure.

### Study parameters

Study parameters included age, gender, body surface area (BSA; [√((height × weight)/3600)], arterial systolic blood pressure (SBP), diastolic blood pressure (DBP), mean arterial pressure MAP, heart rate (HR), pulse pressure (PP), pulse pressure variation (PPV), stroke volume variation (SVV), stroke volume (SV) or cardiac index (CI), Patients were categorized according to age (<55 or ≥55 years) or gender. Dynamic arterial elastance (Ea_dyn_) was defined as the PPV/SVV ratio and used as indicator of arterial tone based on the publication of Monge Garcia et al. [18]. We further evaluated the number of patients with an increase in MAP and CI upon Trendelenburg positioning in subjects with a steady state Ea_dyn_ below or exceeding 0.89, which was defined by Monge Garcia as the value that discriminates between blood pressure unresponsiveness to fluids (<0.89) or responsiveness to fluids (≥0.89). In case of Ea_dyn_ < 0.89 vasopressors are required for increasing the MAP [18].

### Data and statistical analysis

Nexfin CC data were extracted using Frame Inspector (Frame inspector software version 2.3.0.2, BMEYE BV, Amsterdam, the Netherlands) and analyzed using SPSS Statistics version 17.0 (IBM, New York, USA). Results are expressed as mean ± SD or median with interquartile range. Mean hemodynamic values were calculated over a period of 30 s recorded during the initial steady state (baseline) and at four consecutive time frames of 15 s during the first minute in Trendelenburg position and in the neutral supine position following Trendelenburg. Pulse pressure variation (PPV) was defined as the relative variation between the highest (PP_max_) and lowest (PP_min_) pulse pressure divided by the mean of PP_max_ and PP_min_ (PPV (%) = 100 × (PP_max_ − PP_min_)/((PP_max_ + PP_min_)/2)). Stroke volume variation (SVV) was defined as the relative variation between the highest (SV_max_) and lowest (SV_min_) stroke volume divided by the mean of SV_max_ and SV_min_ (SVV (%) = 100 × (SV_max_ − SV_min_)/((SV_max_ + SV_min_)/2)). Changes in hemodynamic parameters upon Trendelenburg positioning (TB start) when compared to steady state values were analyzed using a paired *T* test. The decrease in PPV and SVV upon Trendelenburg positioning was assessed using repeated measures ANOVA (RM).

The ability of the SVV or PPV to predict a relative increase in stroke volume of 10 % or more upon Trendelenburg positioning, which was defined as fluid responsiveness, was assessed with a Receiver Operating Characteristic (ROC) curve. The relative increase in stroke volume was determined from baseline values and the highest value during Trendelenburg positioning. The predictive value of PPV or SVV was expressed as the area under the curve (AUC) with 95 % confidence intervals. The AUC’s for the SVV and PPV were compared using the method described by DeLong et al. [19]. A Mann–Whitney *U* test was performed to determine statistical differences among the difference between PPV and SVV for age and gender, while Pearson correlations was calculated for the relation between different parameters. Frequencies were analyzed using a chi-square test. A *P* value of less than 0.05 was considered as statistically significant.

## Results

### Patient characteristics

The study included 54 patients (30 females/24 males) with an average age of 45 ± 16 years and body mass index of 25.5 ± 3.6 kg/m^2^. We observed no technical failure of the Nexfin device, and retrieved a non-invasive arterial blood pressure waveform in all included patients. After anesthesia induction and endotracheal intubation, baseline hemodynamic parameters estimated 98 ± 16 mmHg (SBP), 62 ± 9 mmHg (DBP), 76 ± 12 mmHg (MAP), 78 ± 13 beats per min (heart rate) and 3.1 ± 0.7 l m^−2^ min^−1^ (CI).

### Changes in hemodynamic parameters during a provoked fluid shift

**Fig. 1:**
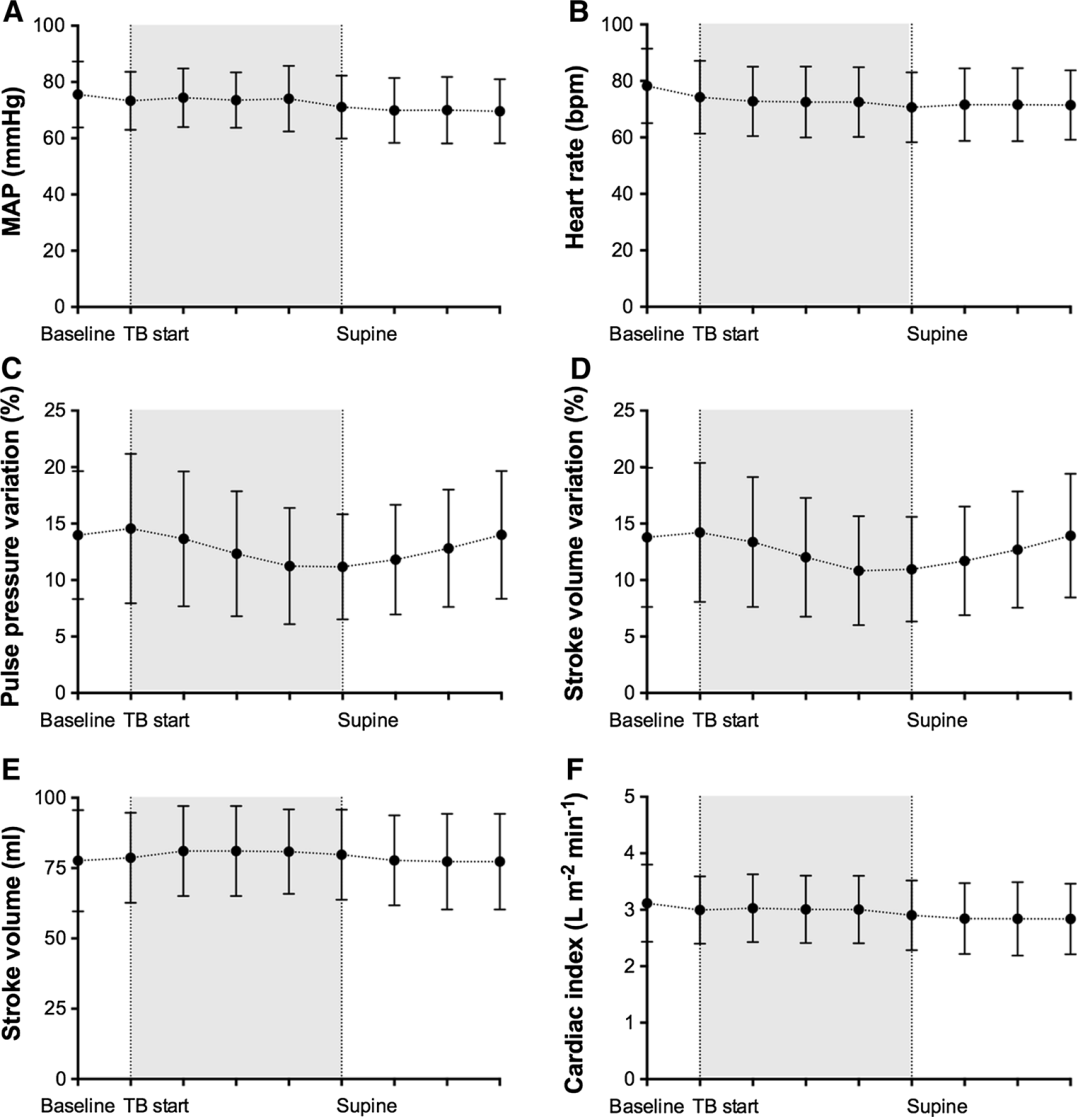
Changes in mean arterial pressure (MAP; **a**), heart rate **b**, pulse pressure variation (PPV; **c**), stroke volume variation (SVV; **d**), stroke volume **e** and cardiac index (CI; **f**) during Trendelenburg (TB) and reversal to neutral supine position. Data represent mean ± SD

Trendelenburg positioning did not induce a change in mean arterial pressure (Fig. 1, panel A), or systolic or diastolic blood pressure (data not shown). Upon Trendelenburg positioning (TB start), the heart rate slightly decreased when compared to steady state (panel B; *P* < 0.001), and remained unaltered throughout the rest of the study period. Both pulse pressure variation (PPV; panel C) and stroke volume variation (SVV; panel D) significantly decreased upon Trendelenburg positioning (RM; both *P* < 0.001), and returned to baseline values after repositioning to supine steady state. While Trendelenburg positioning slightly increased stroke volume (TB start; *P* = 0.02; panel E) when compared to steady state values, cardiac index remained unaltered (panel F). The number of patients with a decrease in SVV or PPV of 3 % or more after 15° Trendelenburg positioning estimated 59 and 56 %, respectively. During Trendelenburg positioning, SVV and PPV decreased by 3.4 ± 2.7 and 3.3 ± 2.8 %, respectively.

**Fig. 2:**
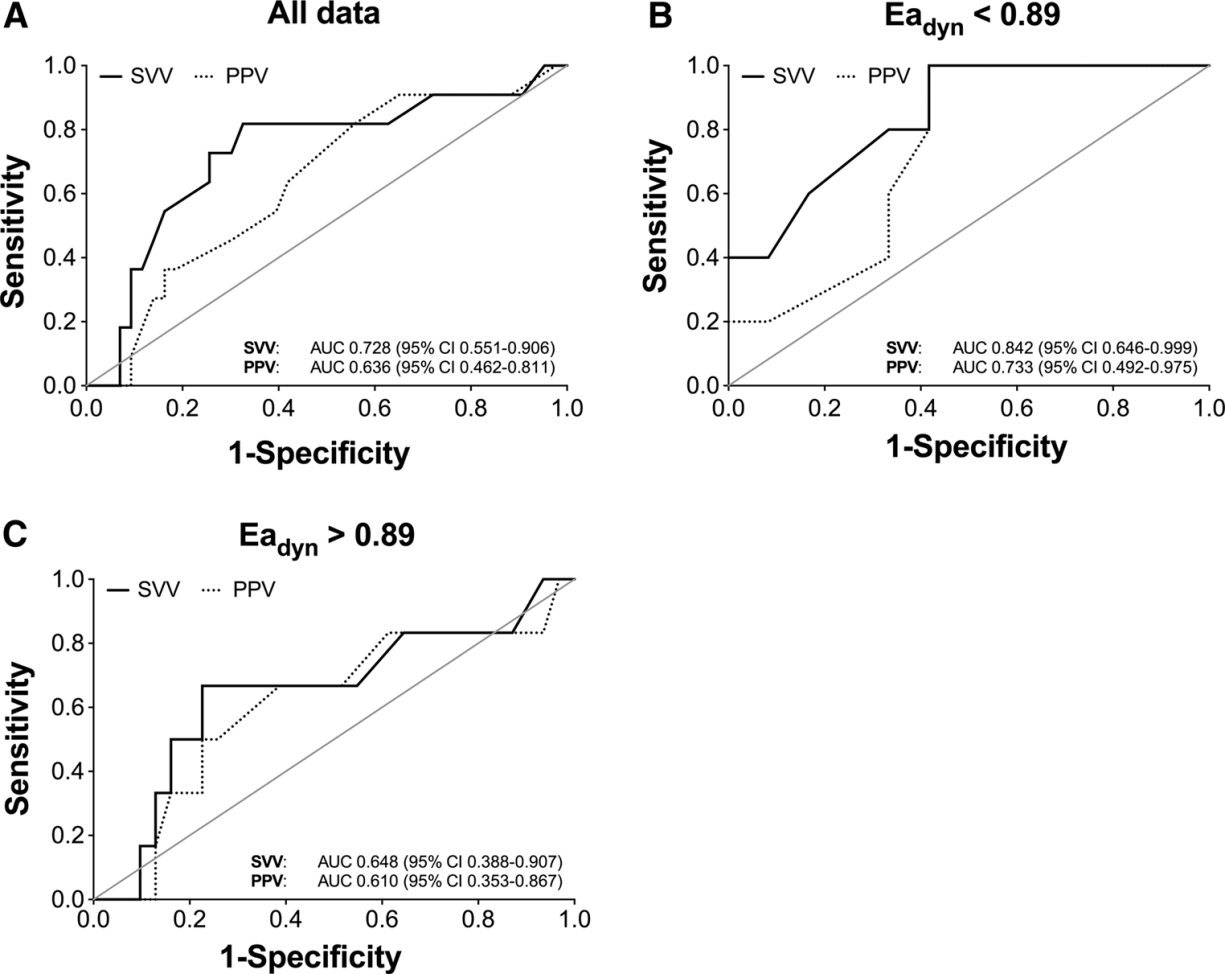
Receiver operating characteristic (ROC) curves to assess the predictive value of the steady state stroke volume variation (**a**; SVV; *straight line*; AUC 0.728 CI 0.551–0.906) and pulse pressure variation (PPV; *dotted line*; AUC 0.636 CI 0.462–0.811) to predict fluid responsiveness defined as an increase in stroke volume of 10 % or more upon Trendelenburg positioning. **b** and **c** show the ROC curves for PPV and SVV in patients with an Ea_dyn_ <0.89 or Ea_dyn_ >0.89, respectively. AUC = area under the curve with 95 % confidence intervals (CI)

Overall, 11 out of 54 patients responded to the fluid challenge as defined by a minimal rise in stroke volume of 10 % during Trendelenburg positioning. The SVV and PPV decreased by 3.7 ± 3.0 and 3.8 ± 3.8 % in case of fluid responsiveness, and 3.3 ± 2.6 and 3.2 ± 2.6 % in patients who did not respond to a fluid challenge, respectively. Figure 2 shows that the SVV (Panel A; AUC 0.728; CI 0.551–0.906; specificity 67 %, sensitivity 82 %) tended to be superior in the prediction of fluid responsiveness compared to PPV (AUC 0.636; CI 0.462–0.811; specificity 44 %, sensitivity 82 %) upon Trendelenburg positioning. There was no significant difference in the AUC’s of the SVV and PPV (*P* = 0.079). Fluid responsiveness was equally present in the group of patients with an Ea_dyn_ < 0.89 or Ea_dyn_ > 0.89 (16 vs. 29 %, respectively; *P* = 0.263). Subdivision of patients according to Ea_dyn_ showed a slight improvement in the AUC for the predictive value of PPV and SVV for fluid responsiveness in subjects with an Ea_dyn_ < 0.89 (Fig. 2, panel B), while the AUC for PPV and SVV converged to lower values in patients with an Ea_dyn_ > 0.89 (panel C).

### Differences between PPV and SVV values

**Fig. 3:**
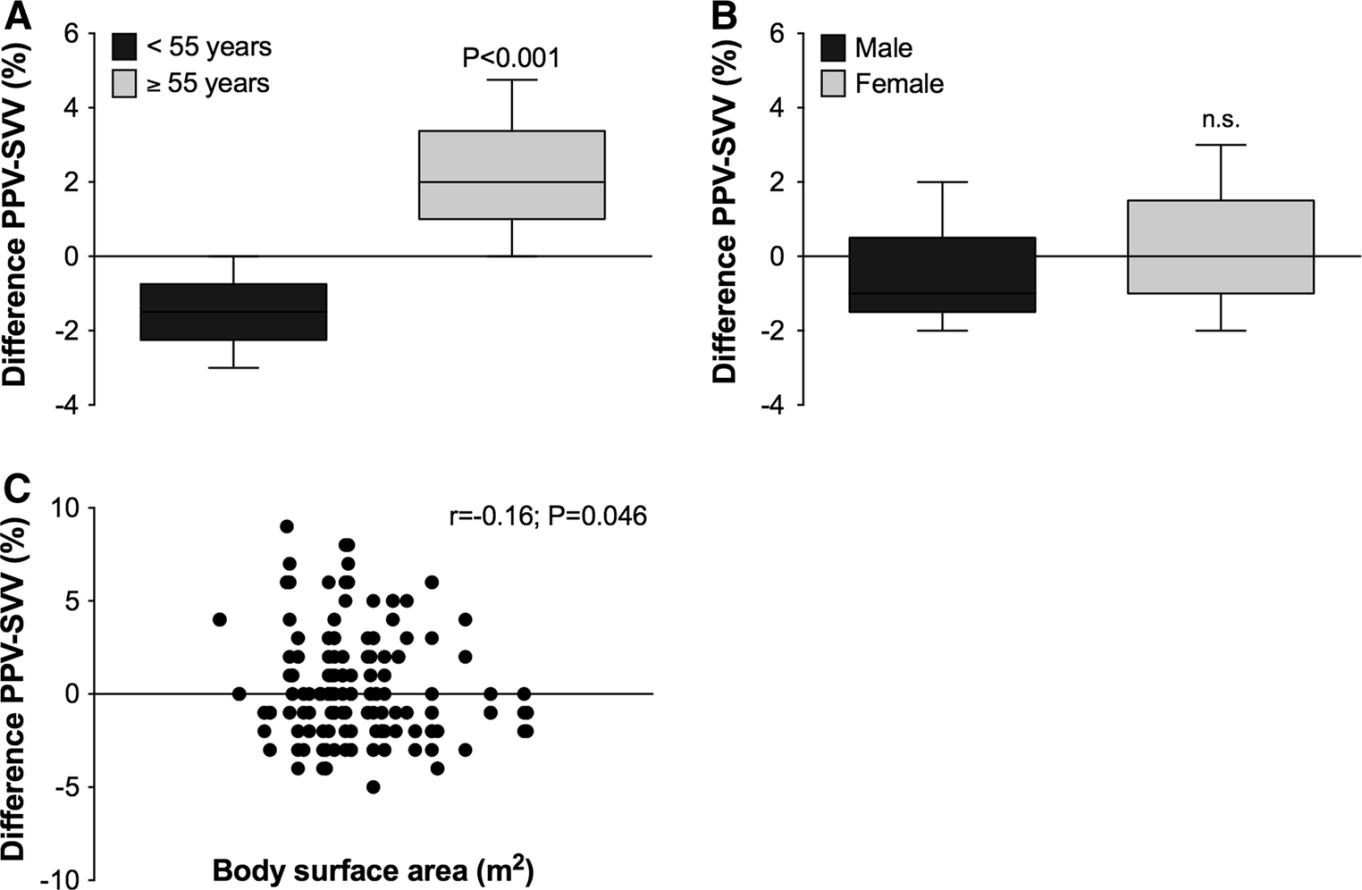
The difference between PPV and SVV as revealed by Bland–Altman analysis was categorized for age (<55 or ≥55 years; **a**), gender (**b**) and body mass index (<25 or ≥25 kg/m^2^; **c**). Data represent mean ± standard deviation.* P* values are shown in the figure panels

Figure 3 shows that the difference between PPV and SVV shifted to negative values (SVV > PPV) for patients younger than 55 years, and to positive values (PPV > SVV) in patients aging 55 years or older (panel A; *P* < 0.001). The PPV–SVV difference was similar among male and female patients (panel B). There was a very small correlation between body surface area and the difference between PPV and SVV (panel C; r = 0.16; *P* = 0.046).

**Fig. 4:**
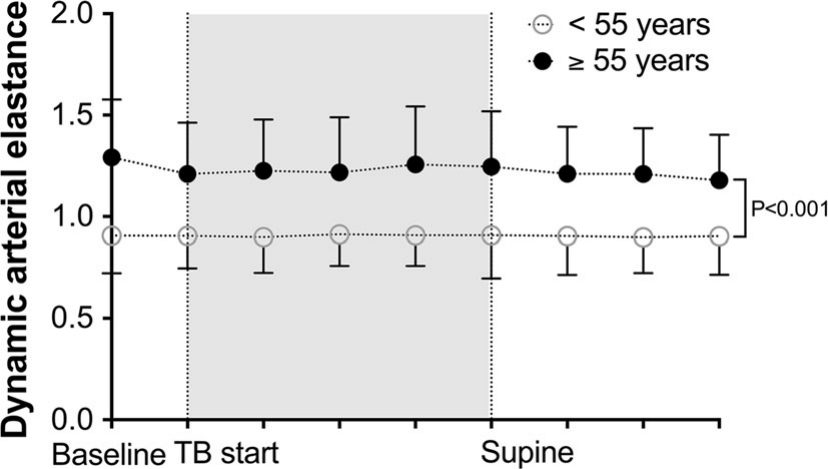
Dynamic arterial elastance (Ea_dyn_) expressed as the ratio between pulse pressure variation and stroke volume variation for patients younger (n = 22) or older (n = 21) than 55 years. Supine = repositioning to supine state. Data represent mean ± standard deviation *P* < 0.001 (repeated measures analysis) for changes in dynamic arterial elastance over time between groups

The dynamic arterial elastance expressed as the PPV/SVV ratio during Trendelenburg positioning and repositioning for patients younger and older than 55 years is shown in Fig. 4. The PPV/SVV ratio was higher for older patients when compared to younger patients (*P* < 0.001; repeated measures analysis). The Ea_dyn_ was further subdivided in values higher or lower than 0.89. In patients with a steady state Ea_dyn_ < 0.89 (all patients with an age <55 years), the MAP and CI increased in 52.9 and 29.4 % of all subjects, respectively, after Trendelenburg positioning. For patients with an Ea_dyn_ ≥ 0.89 (all patients with an age ≥55 years), the MAP and CI increased in 40.5 and 40.5 % of the cases, respectively, after Trendelenburg positioning.

## Discussion

This study shows that the Nexfin non-invasive arterial blood pressure monitor reflects a mild provoked fluid shift after Trendelenburg positioning in mechanically ventilated patients by alterations in the pulse pressure variation (PPV) and stroke volume variation (SVV), while the mean arterial pressure (MAP) and cardiac index (CI) were not sensitive enough to reflect this fluid challenge. While PPV is directly derived from non-invasive arterial blood pressure measurements, the Nexfin SVV is calculated based on the Nexfin CO-trek algorithm, which requires input of patient demographics. The baseline SVV tended to be superior over PPV in predicting fluid responsiveness in our population. Our findings indicate that the PPV and SVV are of additional value to static indices for clinical determination of fluid shifts in anesthetized patients.

In accordance to our study, Rex et al. [20] investigated the effects of Trendelenburg positioning (30°) on hemodynamic parameters and found a decrease in stroke volume variation, but increase in cardiac index following Trendelenburg. Cardiac index had a lower predictive value for fluid responsiveness than SVV [20]. They concluded that SVV is more dominantly influenced by cardiopulmonary effects on the filling state of the patient, while cardiac index is subject to changes in preload, the position at the Frank Starling curve and a baroreceptor-mediated decrease in heart rate following Trendelenburg positioning [20]. We only observed a small decrease in heart rate, while cardiac index remained stable during Trendelenburg positioning based on the increase in stroke volume. In 20 % of the patients, stroke volume increased by 10 % or more, and these patients were indicated as fluid responsive. Although the SVV and PPV had a predictive value that exceeded 0.5, both indices did not reach a high specificity and sensitivity.

Continuous arterial blood pressure monitoring was used for evaluation of ventilation-induced changes in pulse pressure and stroke volume. While the PPV is directly derived from the blood pressure signal, evaluation of the SVV requires an additional algorithm based on pulse contour analysis from arterial blood pressure waveforms [11]. It might be argued that the algorithm required for calculation of the SVV may introduce a measurement bias, as this algorithm requires insight in individual aortic compliance. Although it was previously shown that the Nexfin CO-trek-algorithm that is required for SVV calculations is superior to pulse contour analysis [11], our insight in the differences between arterial blood pressure-based PPV and SVV values is currently limited. Cannesson et al. [21] compared respiratory variations in pulse pressure with SVV using the Vigileo/FloTrac arterial blood pressure device, and found a bias of −1.3 % with a deviation of 2.8 %. In light of this small bias, they concluded that SVV monitoring could serve as alternative for pulse pressure variation, as Vigileo/Flotrac does not allow continuous monitoring of changes in pulse pressure. A second study that focused on a comparison of Ohmeda PPV with Vigileo/FloTrac SVV in surgical patients revealed a bias of −0.70 % between PPV and SVV, which was also small enough to suggest that the PPV could be used in clinical routine [22]. In our study we used one device to simultaneously measure PPV and SVV during Trendelenburg positioning, which does not allow a level of agreement analysis. However, we observed differences in PPV and SVV values, and questioned whether the calculation of SVV based on the arterial blood pressure waveform might be influenced by patient demographics that are required for the Nexfin CO-trek-algorithm. Indeed, we found that the difference between PPV and SVV in younger patients (<55 years) changed from a negative bias to a positive bias in older patients (≥55 years). The Nexfin CO-trek-algorithm uses a fixed estimation of vascular compliance based on gender and age, and stroke volume calculations may therefore reveal an age-dependent effect, resulting in a lower SVV than PPV following a provoked fluid shift in older subjects. Hofer et al. [23] stated the SVV to be superior to PPV for predicting fluid responsiveness from a physiological point of view, since PPV is assumed to be more susceptible to vascular influences than SVV, but our data suggest that the PPV is a more age-independent parameter for the determination of fluid responsiveness using the Nexfin monitor.

Nexfin cardiac index values have proven to be unreliable in studies in critically ill patients, since they included patients with potentially confounding factors due to abnormal vascular tone, peripheral hypoperfusion due to septic shock, or cardiac stunning [24–26]. The present study was performed in a population with normal peripheral perfusion, and previous reports have shown a good level of agreement with thermodilution measurements or transthoracic or esophageal Doppler cardiac index in this population [10, 11, 16, 27]. Bubenek et al. [28] concluded the Nexfin device to have limited accuracy compared with the pulmonary artery catheter, however being able to reliably track cardiac output changes after inducing preload-modifying actions in a post-cardiosurgical population.

We investigated whether the Ea_dyn_ can be used in the decision to administer fluids or vasopressors as previously suggested [18]. We however found that, using Nexfin hemodynamic monitoring, the PPV/SVV ratio as indicator of Ea_dyn_ is different in younger and older subjects. These findings should be considered in light of the assumption that all included patients had a normal preload reserve, which is a prerequisite for Ea_dyn_ measurements. An adequate preload reserve is defined as a normal distensibility of the left ventricle, and is most likely to be normal in our study population that consisted of subjects without diabetes mellitus or cardiovascular diseases. Besides, the proposed Ea_dyn_ threshold for determination of blood pressure sensitivity to fluid loading seemed unreliable in the current population, although we have to emphasize that vasopressor effects were not evaluated in this study.

We used a tidal volume of 8 ml/kg or more, with a positive end expiratory pressure of 5 mmHg in patients with a closed thorax and without arrhythmias in order to reliably measure PPV and SVV [29]. Trendelenburg positioning was performed to provoke a fluid shift in our patients, and we acknowledge that this method induces a weaker response in dynamic indices than a fluid bolus or passive leg raising [30]. The disadvantage of a mild fluid shift is that the subsequent changes in hemodynamic indices might be blurred by the variation in the precision of the Nexfin device. Our study is further limited by the use of data obtained following anesthesia induction, without taking intraoperative fluid shifts and surgical maneuvers into consideration.

In conclusion, we show that Nexfin PPV and SVV reflect changes in the filling state of the patient, and are more sensitive to these fluid changes than mean arterial pressure and cardiac index. In the context of its non-invasive nature, Nexfin may therefore be of clinical value during monitoring of patients subjected to intraoperative fluid shifts. However, there is an age-dependent difference in PPV and SVV, which may be of influence on the choice for the right dynamic indicator for fluid responsiveness.
